# Video Telehealth Occupational Therapy Services for Older Veterans: National Survey Study

**DOI:** 10.2196/24299

**Published:** 2021-04-27

**Authors:** Megan E Gately, Linda Tickle-Degnen, Deborah J Voydetich, Nathan Ward, Keren Ladin, Lauren R Moo

**Affiliations:** 1 Department of Veterans Affairs Geriatric Research Education and Clinical Center Bedford, MA United States; 2 Department of Occupational Therapy Tufts University Medford, MA United States; 3 Department of Psychology Tufts University Medford, MA United States; 4 Department of Veterans Affairs Veterans Affairs Central Office Washington, DC United States; 5 Department of Community Health Tufts University Medford, MA United States; 6 Department of Public Health and Community Medicine Tufts University School of Medicine Boston, MA United States; 7 Department of Neurology Harvard Medical School Cambridge, MA United States

**Keywords:** occupational therapy, telemedicine, health services, older adults

## Abstract

**Background:**

Occupational therapy (OT) is a vital service that supports older adults’ ability to age in place. Given the barriers to accessing care, video telehealth is a means of providing OT. Even within Veterans Health Administration (VHA), a pioneer in telehealth, video telehealth by OT practitioners to serve older adults is not well understood.

**Objective:**

This study examines VHA OT practice using video telehealth with older veterans using an implementation framework.

**Methods:**

A web-based national survey of VHA OT practitioners conducted between September and October 2019 contained a mix of mostly closed questions with some open-text options. The questions were developed using the Promoting Action on Research Implementation in Health Services model with input from subject matter experts. The questions gathered the extent to which VHA OT practitioners use video telehealth with older veterans; are comfortable with video telehealth to deliver specific OT services; and, for those using video telehealth with older veterans, the barriers, facilitators of change, and perceived benefits of video telehealth.

**Results:**

Of approximately 1455 eligible VHA OT practitioners, 305 participated (21.0% response rate). Most were female (196/259, 75.7%) occupational therapists (281/305, 92.1%) with a master’s degree (147/259, 56.8%) and 10 years or fewer (165/305, 54.1%) of VHA OT practice. Less than half (125/305, 41.0%) had used video telehealth with older veterans, and users and nonusers of video telehealth were demographically similar. When asked to rate perceived comfort with video telehealth to deliver OT services, participants using video telehealth expressed greater comfort than nonusers, which was significant for 9 of the 13 interventions: activities of daily living (*P*<.001), instrumental activities of daily living (*P*=.004), home safety (*P*<.001), home exercise or therapeutic exercise (*P*<.001), veteran or caregiver education (*P*<.001), durable medical equipment (*P*<.001), assistive technology (*P*<.001), education and work (*P*=.04), and wheelchair clinic or seating and positioning (*P*<.001). More than half (74/125, 59.2%) of those using video telehealth reported at least one barrier, with the most frequently endorsed being *Inadequate space, physical locations and related equipment*. Most (92/125, 73.6%) respondents using video telehealth reported at least one facilitator, with the most frequently endorsed facilitators reflecting respondent attitudes, including the belief that video telehealth would improve veteran access to care (77/92, 84%) and willingness to try innovative approaches (76/92, 83%).

**Conclusions:**

Most VHA OT survey respondents had not used video telehealth with older veterans. Users and nonusers were demographically similar. Differences in the percentages of respondents feeling comfortable with video telehealth for specific OT interventions suggest that some OT services may be more amenable to video telehealth. This, coupled with the primacy of respondent beliefs versus organizational factors as facilitators, underscores the need to gather clinicians’ attitudes to understand how they are driving the implementation of video telehealth.

## Introduction

### Background

Veterans Health Administration (VHA), the largest integrated health care system in the United States, has been using telehealth since the 1990s to provide care to a broadly dispersed veteran population. VHA provides care to veterans who served in military, naval, or air services. Approximately 60% of US veterans are enrolled in VHA care, including more than 90% of those who incurred a service-related disability. The median age of veterans is 65 years, including a large portion of rural veterans [1-4]. To ensure access to care by veterans regardless of where they live, VHA has undergone a major expansion of telehealth services, including video telehealth, a live, synchronous encounter in which patients and providers are in 2 different locations, as part of the Veterans Affairs (VA) Maintaining Internal Systems and Strengthening Integrated Outside Networks (MISSION) Act, which expands video telehealth into veterans’ homes or another location of choice [5,6]. This represents a dramatic shift from most VHA video telehealth services, which were conducted between 2 clinic locations (eg, large medical centers to community-based clinics). Through strategies including issuing tablets to high-need veterans [7] and developing a dedicated VA videoconferencing app that was compliant with the Health Insurance Portability and Accountability Act and other privacy protections, use of video telehealth increased by 235% in fiscal year 2019, with more than 99,000 veterans using the app from home. More than two-thirds of this increase was for telemental health [8], which has represented the majority of VHA telehealth use since its inception.

Video telehealth for specialty care such as occupational therapy (OT) has historically been underdeveloped; of an estimated 1.5 million total VHA OT encounters in fiscal year 2018, less than 1% were delivered using telehealth [9]. This is despite the integration of telehealth into OT practice being identified as a professional goal [10-12]. Constraining integration is a lack of evidence for OT video telehealth, particularly for older adults. OT plays a key role in supporting older adults’ participation in activities of daily living (ADL) such as bathing and dressing [13], instrumental activities of daily living (IADL) such as medication management and meal preparation [14], and home modifications to increase safety and prevent injury [15]. Although evidence for telerehabilitation is growing [9,16-20], with video telehealth being used for exercise [21-23], recent reviews of telehealth OT highlight a paucity of evidence [24-26]. Furthermore, there are barriers to using technology for older adults, including low technical literacy [27] and some older adults’ preference for telephone [28]. Telephones are inherently limited for OT clinical care because they lack a visual component [29]. Similar to telephone, asynchronous technologies such as mobile health apps and tablet-based apps, which have also been used with older adults [29-32], do not have a live component, which is critical to responding to clients’ needs in the moment. Thus, video telehealth is a complex occupation [33] that involves sophisticated technologies that may be challenging for those with less technical expertise.

Lack of evidence for OT video telehealth has resulted in a gap in knowledge about how best to integrate video telehealth into OT practice in response to the COVID-19 pandemic [9]. Little is known about how such diverse, complex OT interventions will be delivered using video telehealth, specifically to older adults, a population with distinct needs that may make participation in video telehealth more challenging because of decreased hearing, vision, and sensory processing; increased rates of cognitive impairment and reliance on family caregivers; as well as overall lower rates of technology literacy and use. Our own work providing home safety evaluations using video telehealth in dementia care highlighted numerous technological challenges, including inconsistent audio and visual signals [26].

To optimize the integration of video telehealth solutions, various contextual factors must be considered, according to the Promoting Action on Research Implementation in Health Services (PARIHS) framework. PARIHS was developed as an evaluative framework to support the systematic integration of research findings and intervention strategies into clinical care, thereby enhancing the quality and efficacy of health services [34]. Clinical experiences and preferences are central to successful implementation, and lack of clinician knowledge and acceptability is a known barrier to telehealth [35]. Limited data on OT perspectives on telehealth highlight either negative attitudes toward or knowledge gaps about telehealth. OT faculty hold less than positive views of telehealth [36], whereas previously surveyed OTs lack awareness of telehealth strategies [37]. No extant study has examined OT practitioners’ perspectives on the use of video telehealth with older adults.

### Objectives

Given this knowledge gap, this study sought to gather OT practitioners’ experiences with and perspectives on video telehealth to serve older adults. Specifically, we sought to ascertain the extent to which VHA OT practitioners use video telehealth to serve older veterans; VHA OT practitioners’ comfort with video telehealth to deliver specific OT services; and, for those using video telehealth with older veterans, perceived barriers, facilitators of change, and benefits of video telehealth. The aim of this study is to identify barriers and facilitators related to the successful implementation of video telehealth to ensure equitable distribution of this service to older adults.

## Methods

### Participants

**Figure 1:**
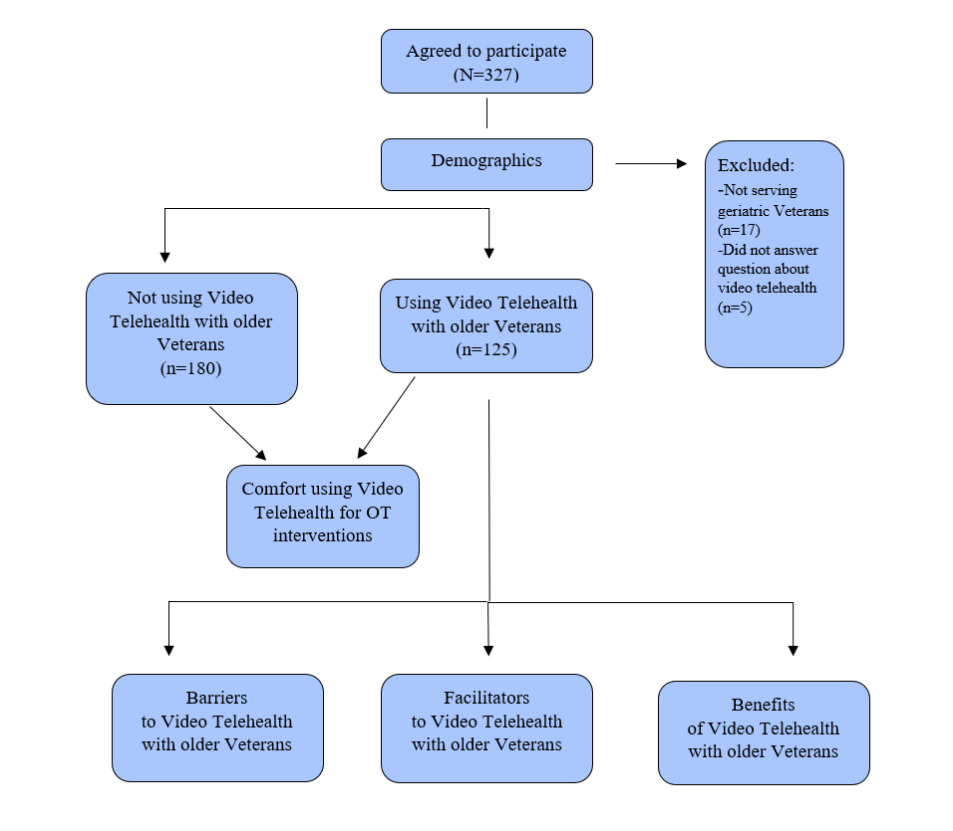
Survey flow diagram. OT: occupational therapy.

To gather data, a survey was conducted with a volunteer sample of OT practitioners enrolled in VHA’s national internal OT email listserv groups from a network of 1243 health care facilities, including 170 VA medical centers and 1063 outpatient sites. Inclusion criteria were being either an occupational therapist or OT assistant and treating older veterans, who were defined as those aged 65 years or older. No other exclusion criteria were included. The survey flow and inclusion criteria are shown in Figure 1.

### Survey Development

Here, we present survey details in accordance with the Checklist for Reporting Results of Internet E-Surveys checklist [38]. Survey items were developed and informed in accordance with the PARIHS framework domains to gather clinicians’ experiences and perceptions of video telehealth to serve older veterans. Specifically, we used a guide developed to operationalize PARIHS concepts [39] as well as our own practice experience with video telehealth and input from VHA stakeholders to develop items that capture contextual factors from the clinicians’ perspective. Items were designed to gather clinicians’ experiences with and perceptions about video telehealth; clinicians’ impressions of veteran experiences, needs, and preferences for video telehealth, to the extent that OT respondents could speak on their behalf; and the characteristics of the local practice, to identify perceived barriers, facilitators, and benefits of video telehealth to serve older veterans.

Items were reviewed for face and content validity by 5 subject matter experts in OT, telehealth, and geriatrics care and revised based on feedback. Before the survey launch, 5 VHA OTs (separate from above) pretested the survey, which involved completing the survey draft via the REDCap (Research Electronic Data Capture) link, followed by cognitive interviews conducted by the first author using predetermined verbal probes [40]. Probes addressed general feedback on survey items and time to administer the survey, which averaged 15 minutes. The survey items were further refined based on the pretesting findings.

The final survey included 17 survey items (Multimedia Appendix 1). Participants were asked if they used or did not use video telehealth with older veterans. Video telehealth was defined as live, synchronous care in which veterans and providers are in 2 locations, connected using Skype-like videoconferencing. Respondents also completed 7 practitioner demographic items, including primary VA medical center and role (eg, occupational therapist or OT assistant). One item addressed respondent comfort with using video telehealth to deliver specific OT interventions. This question included the statement, “We would like to know your level of comfort (that is, the amount of doubt or feelings of stress you feel) about use of video telehealth to deliver OT services at VHA,” and included a list of 13 OT interventions. Interventions included ADL (eg, bathing, dressing, and functional mobility), IADL (eg, meal preparation, financial management, and medication management), and home safety. A complete list of interventions is given in Multimedia Appendix 1. Respondents rated their comfort using video telehealth for each intervention on a 4-point Likert scale, ranging from *not comfortable* to *very comfortable*. An option of *not sure* was provided. We collapsed the 4-point comfort scale into 2 categories—*comfortable* and *not comfortable*—as there were insufficient data to retain the 4 categories. The *not sure* option was excluded, as it was found to be uninterpretable. The comfort item, which was not required, was provided to all respondents whether they used video telehealth or not. Six additional checklist items were completed only by those using video telehealth and addressed the perceived barriers (2 items), facilitators (2 items), and benefits (2 items). A complete list of barriers, facilitators, and benefits is given in Multimedia Appendix 1. Barriers, facilitators, and benefits questions each included options for *other* and *none* and an open-text option whereby respondents could write in additional entries via short open-ended responses. For all checklist items, respondents could select more than one option. Respondents were able to review their answers using the back button. Responses were anonymous; however, as each participant was not sent an individualized survey link, respondents could potentially complete the survey more than once.

### Survey Administration

The survey was conducted in September and October 2019. Practitioners were invited to participate by emailing a survey link to the VHA OT provider listserv and posting on the VHA’s web-based forum for OTs. An anonymous URL link only available on the VA intranet was used. Survey respondents had to be logged into an active VA network account to respond to the survey. The survey was kept open for 4 weeks, with 3 reminder emails and forum posts sent before the survey closed. The email invitation and survey overview specified that participation was voluntary, anonymous, and confidential and that those who agreed to participate agreed to these conditions. Survey data were collected and managed using the REDCap electronic data capture tools hosted at VHA. REDCap is a secure web-based app designed to support data capture for research studies [41]. Survey administration was deemed quality improvement, as it was conducted for VHA organizational purposes. Subsequent analysis of survey data for research purposes was approved by the VA Bedford Health Care System Institutional Review Board.

### Data Analysis

Survey data were exported from REDCap into Excel (Microsoft Corporation) and imported into R for analysis. To statistically examine baseline differences in demographics and differences between perceived comfort for those using video telehealth or not, chi-square or Fisher exact tests (when cell counts were <5) were used for categorical variables and two-tailed *t* tests were used for continuous variables. Statistical significance was set at *P*<.05. For the purposes of analysis, the categorical item, VHA years of practice, was divided into 2 segments of nearly equal size: 10 years or less and 11 years or more. *Prefer not to answer* responses were excluded from analysis, as this option lacked specificity recommended for demographics questions [42]. Rurality geocoding developed by VHA’s Office of Rural Health to estimate the percentage rurality of the catchment area was applied to respondents’ primary medical center.

Short open-ended responses to barriers, facilitators, and benefits items were analyzed using conventional content analysis [43]. Two clinician researchers with experience in telehealth and qualitative analysis (MEG and LRM) repeatedly read entries to determine whether open-ended responses differed from checklist item options and to identify keywords and phrases. These were used to elucidate the given categorical entries and to develop categories for any additional barriers, facilitators, and benefits categories from open-text responses.

## Results

### Overview

Overall, from approximately 1455 eligible VHA OT practitioners, 305 participated (21.0% response rate). The survey flow is shown in Figure 1. Of the 305 respondents, 244 (80.0%) provided complete survey entries, and all entries were included in the analysis regardless of completion.

### Participant Characteristics

Table 1 displays respondents’ demographics. Most respondents were female (196/259, 75.7% of responses), had a master’s degree (147/259, 56.8% of responses), and were occupational therapists (281/305, 92.1% of responses) with 10 years or fewer (165/305, 54.1% of responses) of VHA OT practice. Respondents were from 107 different VA medical centers, the catchment areas of which served a veteran population, which was, on average, 33% rural. Regarding ethnicity, of 258 responses, 16 (6.2%) identified as Hispanic, 197 (76.4%) identified as Not Hispanic or Latino, and 46 (17.8%) preferred not to answer. Regarding race, of 259 responses, 178 (68.7%) identified as White, 50 (19.3%) preferred not to answer, 20 (7.7%) identified as Black or African American, 13 (5.0%) identified as Asian, and 4 (1.5%) identified as American Indian or Alaska Native or Native Hawaiian or other Pacific Islander. Of note, respondents could select more than one racial category. This sample was similar to VHA OT practitioners in terms of years of practice experience, race, ethnicity, and gender, according to the internal administrative VHA data. (VHA data on education were not available.) The sample was considered representative of OT practitioners nationally, as demographics closely aligned with the American Occupational Therapy Association demographics of OT practitioners in terms of gender, race, ethnicity, and education (The Coalition of Occupational Therapy Advocates for Diversity) [44,45].

**Table 1:** Respondents’ characteristics by use of video telehealth with older veterans.^a^

Demographic variables			Using video telehealth (n=125)		Not using video telehealth (n=180)
**Gender, n (%)**					
	Male	12 (12.9)		21 (13.2)	
	Female	70 (75.3)		119 (74.8)	
	Nonbinary	1 (1.1)		2 (1.3)	
	Prefer not to answer	10 (10.7)		17 (10.7)	
**Years of VHA^b^ practice, n (%)**					
	<5	20 (16.0)		55 (30.6)	
	5-10	40 (32.0)		50 (27.7)	
	11-20	46 (36.8)		52 (28.9)	
	21-30	16 (12.8)		19 (10.6)	
	>30	3 (2.4)		4 (2.2)	
**Highest education level, n (%)**					
	Associate’s	4 (4.2)		4 (2.6)	
	Bachelor’s	27 (29.0)		47 (29.5)	
	Master’s	50 (53.8)		93 (58.5)	
	Doctorate	10 (10.8)		11 (6.9)	
	Prefer not to answer	2 (2.2)		4 (2.5)	
**Rural veterans served at respondent’s primary VAMC^c^ (%)**					
	Mean (SD)	34.2 (23.1)		31.5 (21.7)	
	Range	0-82		0-95	

^a^Not all questions were required. Percentages reflect the proportion of respondents who answered the questions.

^b^VHA: Veteran Health Administration.

^c^VAMC: Veterans Affairs Medical Center.

### Utilization of Video Telehealth to Serve Older Veterans

Less than half (125/305, 41.0%) of survey respondents used video telehealth with older veterans. There were no statistically significant differences between respondents using video telehealth and those not using video telehealth according to demographic characteristics. The sample characteristics using video telehealth are shown in Table 1.

### Association Between Comfort Using Video Telehealth for Specific OT Interventions and Use of Video Telehealth

Multimedia Appendix 2 displays comfort using video telehealth to deliver specific OT interventions in 2 categories—*comfortable* and *not comfortable*—by using video telehealth. Respondents who used video telehealth were more likely to express comfort using video telehealth, which was true for 9 out of 13 interventions, except for leisure, social participation, rest and sleep, and sensory or cognitive strategies. More respondents were comfortable with the idea of using video telehealth for these 9 interventions than were not comfortable with them. This was true among both those who had and had not used video telehealth; however, the comfortable versus not comfortable difference was greater for users. Mean comfort ratings with confidence intervals are given in Multimedia Appendix 3.

The 9 interventions showing this statistically significant relationship, with sample sizes for users versus nonusers of video telehealth in parentheses (as this question was not required, respondent totals varied), were ADL (n_user_=69; n_nonuser_=67), IADL (n_user_=66; n_nonuser_=101), home safety (n_user_=78; n_nonuser_=117), home exercise or therapeutic exercise (n_user_=75; n_nonuser_=118), wheelchair clinic or seating and positioning (n_user_=53; n_nonuser_=97), durable medical equipment provision or follow-up (n_user_=80; n_nonuser_=115), veteran and/or caregiver education or training (n_user_=85; n_nonuser_=114), education and work (n_user_=55; n_nonuser_=97), and assistive technology provision or follow-up (n_user_=69; n_nonuser_=101). No significant relationships between comfort and use of video telehealth were found for the interventions of sensory or cognitive strategies (n_user_=52; n_nonuser_=90), social participation (n_user_=52; n_nonuser_=101), leisure (n_user_=52; n_nonuser_=103), and rest and sleep (n_user_=51; n_nonuser_=100).

### Barriers for Those Using Video Telehealth

Table 2 displays a list of organizational barriers and their frequency. The total number of barriers selected by respondents ranged from 1 to 4, with an average of 1.76 per respondent. More than half (74/125, 59.2%) of those using video telehealth encountered at least one barrier. Of 146 total barriers (respondents could select more than one), the most frequently selected barrier was *Inadequate space, physical locations and related equipment*, selected by 50% (37/74) of respondents reporting barriers, whereas lack of leadership support was the least frequent, selected by 8% (6/74) of respondents. More than a quarter (19/74, 26%) indicated that they encountered no barriers. Of the 74 respondents, 23 (31%) provided short, open-text *other comments*, which were then categorized as one of the listed barriers or as novel. Most open-text comments expanded on the barriers selected from the list provided. For example, some described challenges related to technology, such as decreased connectivity or veterans’ lack of technical ability.

**Table 2:** Responses from occupational therapists using video telehealth to the questions “What, if any, barriers have you encountered in adding video telehealth to your practice?” and “What has helped you to add video telehealth to your practice?” (n=74).^a^

Question category		Responses, n (%)
**Barriers**		
	Inadequate space, physical locations, and related equipment	37 (50)
	Delays in process to set up video telehealth (eg, clinic creation and establishing TSA^b^)	35 (47)
	Lack of administrative support (eg, assistance with scheduling and setting up clinics)	26 (35)
	Other	23 (31)
	None	19 (26)
	Lack of leadership support	6 (8)
**Facilitators**		
	Belief that video telehealth with improve veterans’ access to care	77 (83)
	Willingness to try new approaches	76 (82)
	Belief that video telehealth will improve veteran care	62 (67)
	Leadership support	54 (58)
	Administrative support (eg, assistance with scheduling and setting up clinics)	47 (51)
	Adequate space, physical locations, and related equipment	40 (43)
	Other	2 (2)
	None	1 (1)

^a^Items rank ordered by the most frequent barrier or facilitator. Totals may exceed 100%, as respondents could select more than one option. Percentages reflect the number of respondents who selected a given option divided by the number of respondents who answered the question.

^b^TSA: telehealth service agreement.

### Facilitators for Those Using Video Telehealth

Reported facilitators, which included both organizational factors and practitioner beliefs, are given in Table 2. The total facilitators selected ranged from 1 to 6 and averaged 3.89 facilitators per respondent, with only 1 respondent selecting *none*. Most (92/125, 73.6%) respondents using video telehealth reported at least one facilitator. The most frequently endorsed facilitators reflected respondent attitudes, including the belief that video telehealth would improve veterans’ access to care (reported by 77/92, 84% reporting facilitators) and willingness to try innovative approaches (reported by 76/92, 83%). Organizational facilitators, such as leadership support, were reported to a lesser degree. *Adequate space, physical locations and related equipment* was the least selected facilitator, which is in concordance with inadequate space being the top barrier. *Other* and *none* were rarely reported.

### Benefits for Those Using Video Telehealth

Table 3 shows the reported benefits. Most (92/125, 73.6%) of those using video telehealth reported at least one benefit, with total benefits ranging from 1 to 6 and averaging 3.35 per respondent. No respondent selected *none*. Top-ranked benefits related to access, with 94% (87/92) of respondents reporting benefits of remediating veteran distance from the medical center or difficulty getting to the medical center. The impact of video telehealth on efficiency, as indicated by the ability to serve more veterans or to see veterans more often, was reported to a lesser degree (39/92, 42%, and 29/92, 32%, respectively). Short open-ended entries primarily elaborated access benefits, with respondents indicating increased opportunities through video telehealth, such as wheeled mobility specialists, to collaborate with other team members.

**Table 3:** Response to the question “As a practitioner, what benefits do you experience from using video telehealth with Veterans?”^a^

Benefit	Responses, n (%)
I can see veterans who live a distance from VA^b^	87 (94)
I can see veterans who have difficulty coming to VA	87 (94)
I get a view into veterans’ homes	63 (68)
I can see more veterans	39 (42)
I can see veterans more often	29 (32)
Other	7 (8)
None	0 (0)

^a^Item rank is ordered by most frequent benefit. Totals may exceed 100%, as respondents could select more than one benefit. Percentages reflect the number of respondents who selected a given benefit divided by the number of respondents who answered the question.

^b^VA: Veterans Affairs.

## Discussion

### Principal Findings

Most VHA OTs who responded to the survey had not used video telehealth with older veterans, with those using video telehealth demographically similar to those not using video telehealth. Differences in comfort with video telehealth for specific OT interventions suggest that some OT services may be more amenable to video telehealth. This, coupled with our finding that respondent beliefs were more pronounced than organizational factors as facilitators, suggests the importance of clinicians’ attitudes in the implementation of video telehealth.

This is the first study to provide insights into the state of OT video telehealth with older adults, a population of heightened interest because of changing demographics and their increased risk of complications and infections related to COVID-19 [46]. Before COVID-19, older adults were an underserved group for telehealth, as Medicare has until recently [47] been most restrictive regarding telehealth reimbursement [48]. COVID-19 prevention protocols, which prohibited older adults from accessing routine and preventive care in the community, sparked a push to provide home video telehealth services to older adults. This survey was conducted in September and October 2019, approximately 5 months before the shift to virtual care in response to the global pandemic. Thus, the perspectives of early adopters of video telehealth, that is, those who integrated video telehealth into their practice before the urgent need to do so because of COVID-19, presents an unbiased perspective on the use of video telehealth [29]. One of our contributions is to provide evidence relevant to building capacity to support a more robust and rapid uptake of video telehealth by OT practitioners. As such, we offer considerations for OT delivery of video telehealth for older adults.

### Considerations for OT Practitioners

Although most respondents were not, at the time of the survey, using video telehealth with older adults, users and nonusers were demographically similar. Given that the highest rated facilitators to and benefits of video telehealth by users included clinicians’ attitudes toward video telehealth, such as the belief that video telehealth would increase access to care, emphasis on perceived benefits could help encourage OT practitioners hesitant to try video telehealth. However, we did not ask those using video telehealth about attitudinal barriers, such as perceived harm or negative impact of video telehealth in terms of decreased privacy or limitations of what can be clinically done in video telehealth. Thus, it is difficult to draw further conclusions about clinicians’ attitudes toward video telehealth from these data.

Regarding respondent comfort using video telehealth for specific areas of OT practice, differences between users and nonusers indicate that using video telehealth may enhance comfort with video telehealth. However, the causal relationship between respondent comfort and use of video telehealth is not clear, that is, Does the use of video telehealth enhance comfort or do those who are more comfortable with the technology opt to use video telehealth? This relationship should be examined in future studies.

Interventions receiving higher ratings of *not comfortable* with video telehealth suggest potential practitioner knowledge gaps about certain areas of OT practice, incongruity between practice and application in video telehealth, and potential limitations of video telehealth. Respondents were less comfortable with the use of video telehealth for sensory or cognitive strategies. This warrants further study, as it is not clear (as specific intervention examples were not provided) what sensory or cognitive strategies respondents were thinking about when they answered this question. Rest and sleep was another practice area that had higher uncomfortable ratings. As a newer area of OT practice [49], there is a dearth of evidence in this area; therefore, clinicians may be less aware of this intervention in general.

In addition, lower comfort for leisure and social participation is noteworthy, given the strongly established role of OT in these areas [14]. Several interventions telehealth users indicated they were comfortable using video telehealth for involved potentially billable or chargeable items, such as provision of durable medical equipment like wheelchairs or walkers. Given this, the potential influence of cost and reimbursement on choice of intervention also warrants further study. Although OT’s emphasis on participation and function is increasingly recognized as important in the prevention and management of chronic conditions, it is not always supported by payment systems that prioritize symptom-based medical treatment [50].

The findings suggesting that certain OT interventions may be more amenable to a video telehealth platform than others warrant further investigation as to clinician decision making around video telehealth. Both those using video telehealth and those not yet using it felt comfortable with the idea of using video telehealth to provide veteran or caregiver education and training. This may reflect either increased comfort with the use of video telehealth to support interventions relying primarily on verbal engagement or the ubiquity of educational strategies to accompany OT interventions. Relatedly, high percentages of feeling comfortable in using video telehealth for home safety is an interesting finding, given that video telehealth home safety evaluations are complex and may require a caregiver or the patient to ambulate through the home while carrying a portable computing device [5]. Similarly, interventions some respondents were less comfortable using video telehealth for, such as assisting with ADL, may require veterans to move throughout the home (eg, transfer in and out of the bathtub and standing at a kitchen counter), which raises safety concerns. Thus, it is important to gather the perceived drawbacks of video telehealth, including poor audio or video quality, lack of comfort with technology, and safety or privacy concerns.

### Considerations for Older Adults

Given that older adults may have less confidence in operating technology and more mobility limitations, OT interventions delivered through video telehealth, particularly more dynamic interventions such as home safety evaluations, should be optimized to meet older adults’ needs. Identifying strategies to train and prepare veterans to participate in OT-delivered video telehealth (eg, how to take measurements during a home safety evaluation or how to position the camera to allow for a full-body view when observing functional mobility) may facilitate the implementation of video telehealth. In addition, certain populations may have complex care needs, which hamper their ability to participate in video telehealth. Caregiver assistance, particularly for adults who have cognitive impairment or are at risk of falls, may also be needed. Promoting eHealth literacy and co-designing interventions to match technology with older users’ needs will optimize telehealth delivery [51,52].

Perceived benefits, which primarily focused on increased access, corroborate VHA’s organizational mission to use video telehealth to increase access to care. Access was partly related to travel distance; however, open-ended responses suggested that access was more broadly conceptualized to include the ability for more timely care and for more care coordination. For example, practitioners noted that video telehealth allowed them to involve different members of the care team. Older adults often manage multiple chronic conditions that require ongoing intervention by several clinicians. Therefore, video telehealth may increase opportunities for interdisciplinary collaboration to address care needs. This may be even more relevant at times such as during the global pandemic when video telehealth is virtually the only option for face-to-face care. Similarly, these findings raise factors relevant to health care systems that aim to integrate video telehealth OT services.

### Considerations for Health Care Systems

Given the dynamic nature of many OT interventions, an important organizational consideration is the inclusion of technical support for both OTs and older adults. Technical support as an organizational component of video telehealth may be more critical for OT than other, more stationary video telehealth encounters. Mental health video telehealth, for example, consists of mostly verbal exchange, whereas OT interventions may involve veterans working on a cooking task in the kitchen or transferring in and out of the bathtub. This raises potential problems around bandwidth and lost visual or audio that may require the involvement of technical support, in addition to the aforementioned safety concerns.

Barriers and facilitators reveal additional organizational considerations in the delivery of OT services using video telehealth, beyond the aforementioned need for technical support. Lack of physical space (the most frequent barrier and least reported facilitator) may reflect the fact that OTs are often treating in shared spaces such as rehabilitation gyms, unlike mental health clinicians who usually have private offices. This highlights the need to consider infrastructure and privacy in the implementation of video telehealth for OT services; however, allowing practitioners to deliver video telehealth from home would lessen space demands. This study also has implications for clinician education and training to ensure that interprofessional trainees are prepared to offer telehealth to older adults [53,54]. Of note, VHA conducts the largest medical education training program in the United States [55], providing an opportunity to train the next generation of clinicians in telehealth delivery.

### Limitations

This study had several limitations. Regarding survey design, we did not ask practitioners using video telehealth to reflect on barriers such as potential harm, safety risks, disruptions related to video telehealth, increasing workload, or necessary time and training to familiarize themselves with technology, which limits the scope of our findings. As we cannot demonstrate causality between comfort and use of video telehealth, more in-depth surveys or qualitative interviews with OTs may elucidate perceived primary causal issues for comfort as well as perceived barriers and facilitators. The lack of description for certain OT interventions listed in the survey (eg, sensory or cognitive strategies) results in difficulty interpreting some comfort ratings. Nonrespondent bias may also constrain generalizability, as practitioners may have felt pressured to participate or those with a strong interest may have been more likely to participate in the survey. We did not collect data on age, and although years of practice is informative, it is not a proxy for age. Finally, we did not ask whether video telehealth was conducted into the home or between major medical centers and satellite clinics, thereby limiting what conclusions can be drawn regarding video to home, a main telehealth strategy in the post-COVID-19 landscape.

### Implications for Practice

On the basis of our findings, the following are some key implications for implementation of video telehealth in delivering OT services to older adults. Implications reflect the myriad contextual factors vital to ensuring that video telehealth meets the needs of both OT clinicians and patients:

1. Perspectives of early OT adopters of video telehealth, including perceived facilitators, may inform those not yet using video telehealth.
2. The benefit of video telehealth in increasing access to care may encourage increased use of video.
3. Gathering practitioner decision making around the use of video telehealth for specific OT interventions will optimize delivery to clients who face access barriers, increasing the reach of extant providers while potentially saving resources such as clinic space.
4. OT practitioners may have unique infrastructure needs, including dedicated private spaces and need for technical support, in the provision of services using video telehealth.

### Conclusions

Video telehealth with older adults as a service delivery model is rapidly expanding, with VHA at the forefront. Early adoption of video telehealth by VHA OT practitioners appears to be driven, in some measure, by clinician experiences and attitudes; however, institutional barriers remain. As the pandemic offered a model of veterans and some clinicians participating in video telehealth from their own homes, institutional barriers such as limited space may be less of a concern in the post-COVID era. Expansion of video telehealth to deliver services to older adults will involve identifying ways to maximize the video telehealth platform through adaptation and tailoring of interventions to provide client-centered care. There is a need for more evidence on video telehealth OT strategies for older adults, which COVID-19 and resulting OT rapid practice change may expedite.

## Appendix

Multimedia Appendix 1 Survey questions.

Multimedia Appendix 2 Comfort with video telehealth for occupational therapy services for video users and nonusers.

Multimedia Appendix 3 Comfort ratings by use of video telehealth.

## Abbreviations

ADL: activities of daily living
IADL: instrumental activities of daily living
MISSION: Maintaining Internal Systems and Strengthening Integrated Outside Networks
OT: occupational therapy
PARIHS: Promoting Action on Research Implementation in Health Services
REDCap: Research Electronic Data Capture
VA: Veterans Affairs
VHA: Veterans Health Administration
