# Supplementary material for: Video Telehealth Occupational Therapy Services for Older Veterans: National Survey Study
Source: JMIR Rehabil Assist Technol. 2021 Apr 27;8(2):e24299. doi: 10.2196/24299 (PMC8114160; doi:10.2196/24299)
Supplement: Multimedia Appendix 1 [file rehab_v8i2e24299_app1.docx]

Survey Questions

*Required (see additional notes below)

Demographics

1. *Do you agree to participate? (Yes/No)
2. *In what VA facility do you currently work? (List of VA Medical Centers)
3. *What is your role? (Occupational Therapist; Occupational Therapy Assistant)
4. About how many years you been a VHA OT practitioner? (Less than 5-10; 11-20; 21-30; More than 30)
5. What is your highest level of education? (Associate's degree; Bachelor's degree; Master's degree; Doctorate degree; Prefer not to answer)
6. Please select your ethnicity. (Hispanic or Latino; Not Hispanic or Latino; Prefer not to answer)
7. Please indicate your sex. (Male; Female; Non-binary; Prefer not to answer)
8. Please select your race. Select all that apply. (American Indian or Alaska Native; Asian; Black or African American; Native Hawaiian or Other Pacific Islander; White; Prefer not to answer.)

Clinic population and care delivery

1. *Do you currently treat Veterans OLDER than 65? (Yes/No)
   1. Required of all
2. *Do you currently use VIDEO TELEHEALTH with Veterans OLDER than 65? (Yes/No)
   1. Required of those who selected Yes to currently treating Veterans over 65

Comfort using video telehealth to deliver OT services

1. Please indicate your comfort (Not comfortable at all; Somewhat comfortable; Comfortable; Very Comfortable; Not Sure) with VHA OT practitioners using video telehealth with Veterans OF ANY AGE to support the following:
   1. ADL (Bathing, dressing, functional mobility, etc.)
   2. IADL (Meal prep, financial management, medication management, etc.)
   3. Home safety
   4. Sensory and/or cognitive
   5. Veteran and/or caregiver education or training
   6. Social participation
   7. Leisure
   8. Home exercise program/therapeutic exercise
   9. Wheelchair clinic/seating and positioning
   10. Durable Medical Equipment provision/followup
   11. Rest and sleep
   12. Education and Work (supporting participation in education or work-related activities)
   13. Assistive Technology provision/follow-up
2. Barriers to Video Telehealth: What, if any, barriers have you encountered in adding video telehealth to your practice?
   1. Lack of leadership support
   2. Inadequate space, physical location and/or related equipment
   3. Lack of administrative support (e.g., assistance with scheduling, setting up clinics, etc.)
   4. Delays in process to set up video telehealth (e.g., clinic creation, establishing TSA)
   5. Other
   6. None
3. Please feel free to describe barriers to adding video telehealth, in addition to above. (Optional.) (open text)
4. Facilitators to Video Telehealth: What has helped you to add video telehealth to your practice? Select all that apply, or select "None."
   1. Leadership support
   2. Adequate space, physical locations and related equipment
   3. Administrative support (e.g., assistance with scheduling, setting up clinics, etc.)
   4. Belief that video telehealth will improve veteran care
   5. Belief that video telehealth will improve veteran ACCESS to care
   6. Willingness to try new approaches
   7. Other
   8. None
5. If there are additional factors that have helped you to add video telehealth to your practice, please describe them here. (open text)
6. Benefits of Video Telehealth: As a practitioner, what benefits do you experience from using video telehealth with veterans? Select all that apply, or "none."
   1. I can see more veterans
   2. I can see veterans who live a distance from VA
   3. I can see veterans who have difficulty coming to VA
   4. I get a view into veterans' homes
   5. I can see veterans more often
   6. Other
   7. None
7. If you experience additional benefits other than above, feel free to write them in here. (open text).
