# Supplementary figures and images for: Video Telehealth Occupational Therapy Services for Older Veterans: National Survey Study
Source: JMIR Rehabil Assist Technol. 2021 Apr 27;8(2):e24299. doi: 10.2196/24299 (PMC8114160; doi:10.2196/24299)

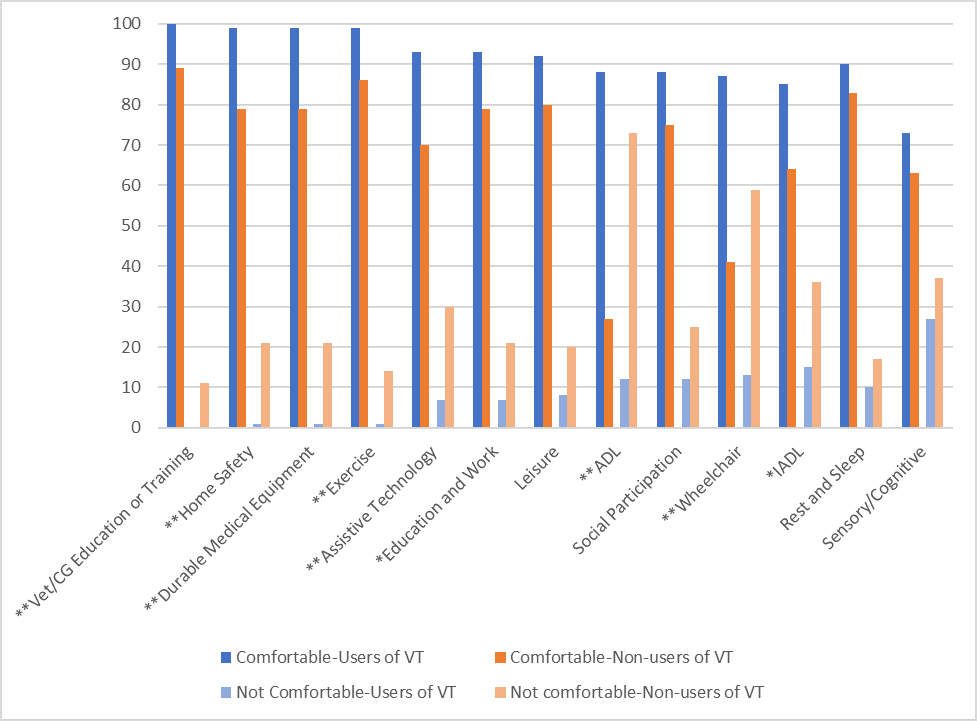

Supplement: Multimedia Appendix 2 [file rehab_v8i2e24299_app2.png]
