# Supplementary material for: Video Telehealth Occupational Therapy Services for Older Veterans: National Survey Study
Source: JMIR Rehabil Assist Technol. 2021 Apr 27;8(2):e24299. doi: 10.2196/24299 (PMC8114160; doi:10.2196/24299)
Supplement: Multimedia Appendix 3 [file rehab_v8i2e24299_app3.docx]

Appendix 3. Comfort ratings by use of video telehealth.^a^

|  |  | VT | Users |  |  | VT | Non-Users |  |
| --- | --- | --- | --- | --- | --- | --- | --- | --- |
|  | N | Mean | 95% Lower Bound | 95% Upper Bound | N | Mean | 95% Lower Bound | 95% Upper Bound |
| **Vet/CG Education and Training** |  |  |  |  |  |  |  |  |
|  | 85 | 1.00 | 1.00 | 1.00 | 114 | 0.89 | 0.83 | 0.95 |
| **Home Safety** |  |  |  |  |  |  |  |  |
|  | 78 | 0.99 | 0.96 | 1.02 | 117 | 0.79 | 0.71 | 0.87 |
| **Durable Medical Equipment** |  |  |  |  |  |  |  |  |
|  | 80 | 0.99 | 0.97 | 1.00 | 115 | 0.79 | 0.71 | 0.87 |
| **Home Exercise/Therapeutic Exercise** |  |  |  |  |  |  |  |  |
|  | 75 | 0.99 | 0.96 | 1.00 | 118 | 0.86 | 0.80 | 0.92 |
| **Assistive Technology** |  |  |  |  |  |  |  |  |
|  | 69 | 0.93 | 0.87 | 0.99 | 101 | 0.70 | 0.61 | 0.79 |
| **Education and Work** |  |  |  |  |  |  |  |  |
|  | 55 | 0.93 | 0.86 | 1.00 | 97 | 0.79 | 0.71 | 0.87 |
| **Leisure** |  |  |  |  |  |  |  |  |
|  | 52 | 0.92 | 0.84 | 1.00 | 103 | 0.80 | 0.72 | 0.88 |
| **ADL** |  |  |  |  |  |  |  |  |
|  | 69 | 0.88 | 0.80 | 0.96 | 67 | 0.27 | 0.16 | 0.38 |
| **Social Participation** |  |  |  |  |  |  |  |  |
|  | 52 | 0.88 | 0.79 | 0.97 | 101 | 0.75 | 0.66 | 0.84 |
| **Wheelchair** |  |  |  |  |  |  |  |  |
|  | 53 | 0.87 | 0.78 | 0.96 | 97 | 0.41 | 0.31 | 0.51 |
| **IADL** |  |  |  |  |  |  |  |  |
|  | 66 | 0.85 | 0.76 | 0.94 | 101 | 0.64 | 0.54 | 0.74 |
| **Rest and Sleep** |  |  |  |  |  |  |  |  |
|  | 51 | 0.90 | 0.82 | 0.98 | 100 | 0.83 | 0.76 | 0.90 |
| **Sensory/Cognitive** |  |  |  |  |  |  |  |  |
|  | 52 | 0.73 | 0.61 | 0.85 | 90 | 0.63 | 0.53 | 0.73 |

^a^Mean comfort ratings by use of video telehealth, including 95% confidence intervals. For a list of unabbreviated

interventions, see Appendix 1. Survey Questions.
